# The Staphylococcus aureus CidA and LrgA Proteins Are Functional Holins Involved in the Transport of By-Products of Carbohydrate Metabolism

**DOI:** 10.1128/mbio.02827-21

**Published:** 2022-02-01

**Authors:** Jennifer L. Endres, Sujata S. Chaudhari, Xinyan Zhang, Janani Prahlad, Shu-Qi Wang, Lily A. Foley, Sorin Luca, Jeffrey L. Bose, Vinai C. Thomas, Kenneth W. Bayles

**Affiliations:** a Center for Staphylococcal Research, Department of Pathology and Microbiology, University of Nebraska Medical Center, Omaha, Nebraska, USA; b Department of Microbiology, Molecular Genetics and Immunology, University of Kansas Medical Center, Kansas City, Kansas, USA; University of Illinois at Chicago

**Keywords:** *Staphylococcus aureus*, holin, membrane protein, pyruvate, transporter

## Abstract

The Staphylococcus aureus *cidABC* and *lrgAB* operons encode members of a well-conserved family of proteins thought to be involved in programmed cell death (PCD). Based on the structural similarities that CidA and LrgA share with bacteriophage holins, we have hypothesized that these proteins function by forming pores within the cytoplasmic membrane. To test this, we utilized a “lysis cassette” system that demonstrated the abilities of the *cidA* and *lrgA* genes to support bacteriophage endolysin-induced cell lysis. Typical of holins, CidA- and LrgA-induced lysis was dependent on the coexpression of endolysin, consistent with the proposed holin-like functions of these proteins. In addition, the CidA and LrgA proteins were shown to localize to the surface of membrane vesicles and cause leakage of small molecules, providing direct evidence of their hole-forming potential. Consistent with recent reports demonstrating a role for the *lrgAB* homologues in other bacterial and plant species in the transport of by-products of carbohydrate metabolism, we also show that *lrgAB* is important for S. aureus to utilize pyruvate during microaerobic and anaerobic growth, by promoting the uptake of pyruvate under these conditions. Combined, these data reveal that the CidA and LrgA membrane proteins possess holin-like properties that play an important role in the transport of small by-products of carbohydrate metabolism.

## INTRODUCTION

The control of bacterial lysis induced by bacteriophage infection is a highly coordinated process that is both elegant and simplistic in nature. This precisely timed event is controlled primarily by two proteins: (i) a murein hydrolase (or endolysin) that specifically degrades the cell wall of the infected cell and (ii) a small membrane-associated protein, termed a “holin,” that controls the access or activity of the murein hydrolase to the peptidoglycan (1–3). The latter has the unique ability to function as a biological timer, delaying peptidoglycan degradation so that bacteriophage assembly can be completed before lysis ensues (4–7). The holin, thus, functions as a master regulator of cell death and lysis akin to the regulatory control of programmed cell death (PCD) in more complex eukaryotic organisms (8, 9). Surprisingly, the most well-studied form of PCD in these organisms (termed “apoptosis”) is also controlled by small membrane-associated proteins (the Bcl-2 family) that target mitochondria (10, 11). Indeed, studies revealed that these proteins functionally complement holin-defective bacterial strains, causing rapid cell lysis (8). Based on these results, it was hypothesized that members of the Bcl-2 family of proteins are functional holins (8) and that the processes underlying the control of apoptosis evolved from bacteria (9, 12).

Our understanding of bacterial PCD has also been shaped by the study of proteins proposed to possess holin-like activity, specifically through investigations of the Staphylococcus aureus CidA and LrgA proteins, which share many characteristics in common with holins. These proteins are also small, they contain two or three transmembrane domains, and their C-terminal domain is highly charged while containing a polar N-terminus (13). In addition to these structural similarities, the CidA and LrgA proteins have also been shown to form dimers and oligomerize into high-molecular-mass structures *in vitro* that are dependent on cysteine disulfide bonds, similar to the S105 holin (14, 15). Early investigations revealed that *cid* and *lrg* mutations caused alterations in murein hydrolase activity and autolysis, consistent with their structural similarities to holins (13, 16). These studies led to the hypothesis that the *cid*/*lrg* family of genes, which are well-conserved in bacteria, as well as in the *Archaea* and plants, are important regulators of PCD (9, 17, 18). Subsequently, the biological role of these genes was associated with biofilm development, a multicellular context in which PCD could provide a benefit to the population (17).

More recently, the genes contained within the *cidABC* operon have been shown to play an important role in regulating cell death during overflow metabolism (19, 20). The CidA and CidB proteins have opposing effects on CidC, a pyruvate:menaquinone oxidoreductase (21), which results in alterations in the extracellular acetate and acetoin levels (19), suggesting that *cidA* and *cidB* encode transport proteins involved in the secretion of these two metabolic by-products (19). Consistent with this was the finding that plant homologs of the *cidAB* and *lrgAB* genes (the Arabidopsis thaliana *PLGG1* gene, also referred to as At*LrgB*) are required for the transport of glycerate and glycolate, two small carbohydrate intermediates important in the Calvin cycle of photosynthesis (22). In addition, it has been shown that the *lrgAB* operons of Bacillus subtilis and Streptococcus mutans are required for pyruvate transport (23–26). The B. subtilis *lrgAB* operon is induced as the amount of extracellular pyruvate increases and mutations within the operon exhibit growth defects when grown on media with pyruvate as primary carbon source. Also, the B. subtilis *lrgAB* operon appears to regulate the intracellular levels of pyruvate as it can also act as an exporter under conditions that promote the accumulation of this metabolite in the cytoplasm (23). Similarly, although S. mutans is unable to grow in media with pyruvate as the sole carbon source, introduction of a *lrgAB* mutation resulted in the inability to import pyruvate once the cultures reached stationary phase when other primary carbon sources have been consumed (25).

In the current study, we use genetic and biophysical approaches to provide further evidence that both CidA and LrgA are functional holins. In addition, we demonstrate that the *lrgAB* operon is involved in pyruvate import under low-oxygen conditions, in contrast to the *cidABC* operon, which was previously shown to affect the export of acetate and acetoin. These studies suggest that the *cidABC* and *lrgAB* operons play a dual role, one as an effector of cell death and the other as transporters of metabolic by-products of carbohydrate metabolism.

## RESULTS

### Functional complementation using *cidA* and *lrgA*.

**FIG 1:**
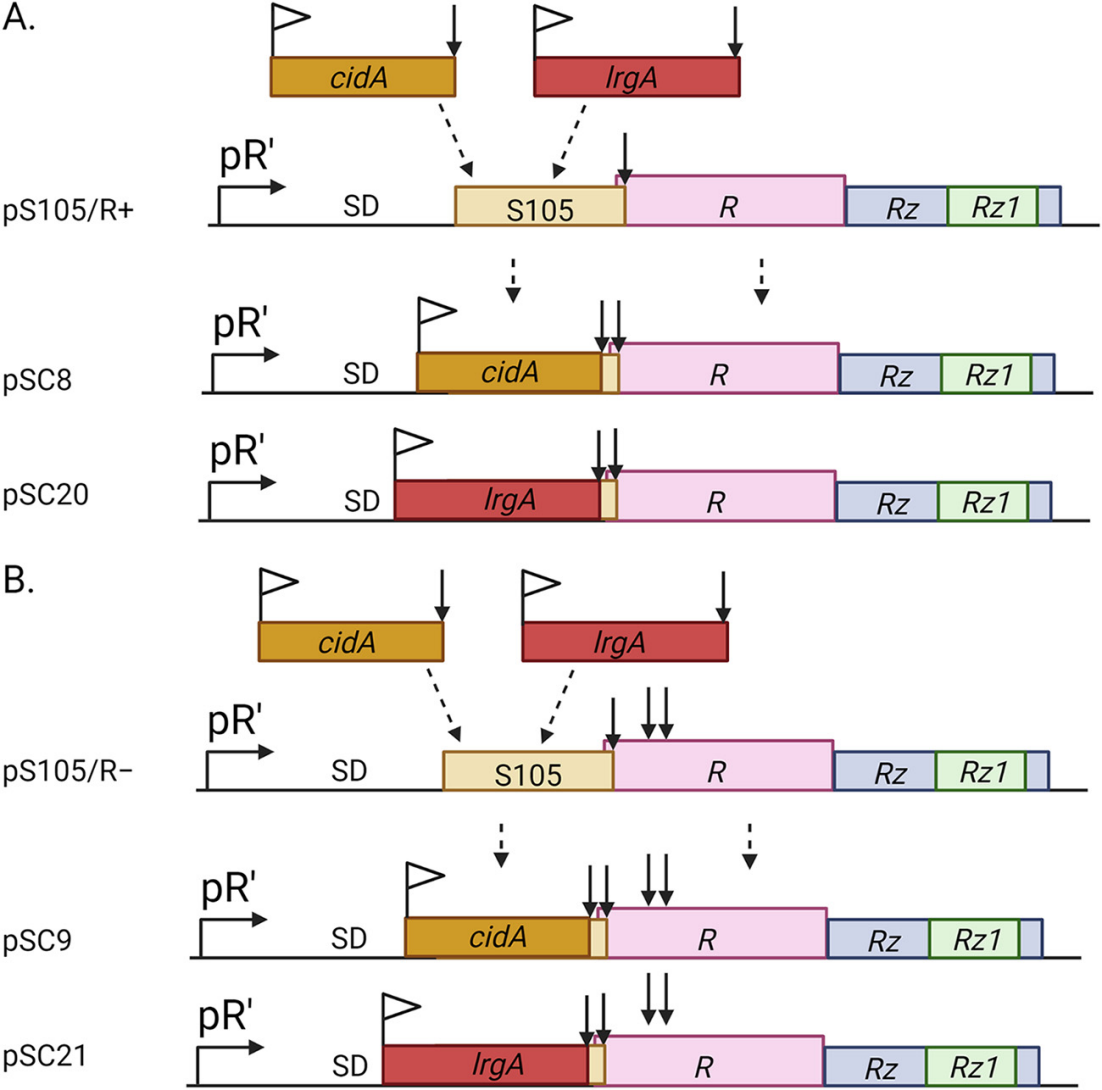
Schematic representation of bacteriophage lambda lysis cassette. The gene encoding S105 holin (X) was replaced with either *cidA* or *lrgA* in (A) pS-FX/R^+^, which expresses endolysin, or in (B) pS-FX/R^−^, which has a stop codon (arrows) introduced so no functional endolysin is produced. pR′, λ late promoter; SD, Shine-Dalgarno sequence; X, S105; R, endolysin and accessory proteins Rz and Rz1.

Although holins are manifested in a variety of different forms, confirmation of holin-like activity can be achieved using the Escherichia coli ΔSR/pS105 holin expression system developed by Smith et al. (27). The plasmid pS105 contains a “λ-lysis cassette” (Fig. 1), encoding a holin (S105), R protein (an endolysin) and the i- and o-spanin proteins Rz and Rz1, respectively. These proteins are products of the λ genes transcribed from the promoter pR′ and are required for lysis and release of newly formed bacteriophage within the cytoplasm of the infected cell. The E. coli ΔSR cells maintain an inducible λ prophage that is lysis defective due to deletion of both *S* (S105) and *R* genes. Following thermal induction, the prophage supplies the late gene activator Q, required for transactivation of the pR′ promoter on pS105, and thus, allows expression of genes within the lysis cassette. The coupled expression of the S105 holin and endolysin following thermal induction causes lysis of the E. coli ΔSR cells, which can be measured spectrophotometrically. Given that lysis is holin dependent, the ability of a heterologous gene to replace the lysis-inducing capacity of S105, therefore, provides a simple way to assess whether this gene encodes a protein with holin-like activity.

**FIG 2:**
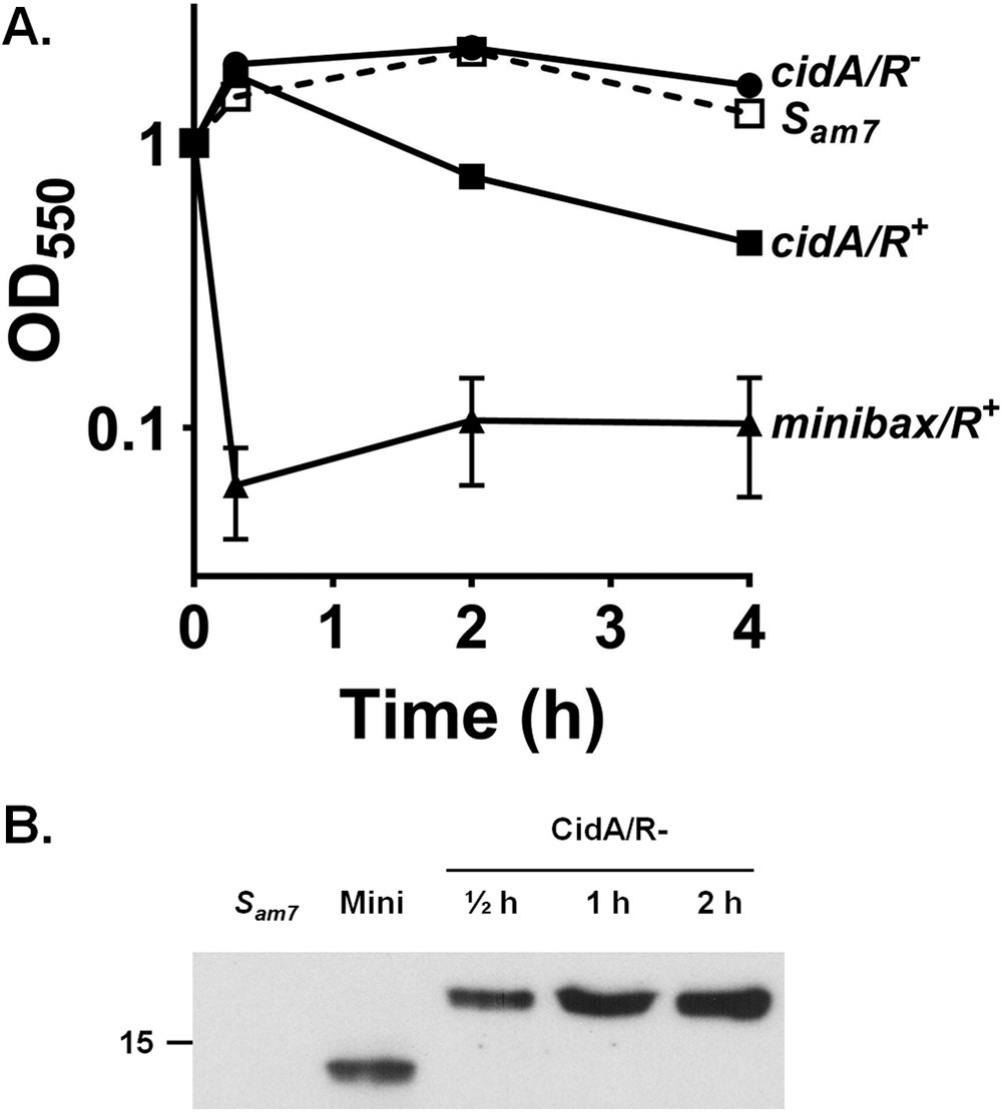
CidA protein exhibits holin-like properties. (A) Cell lysis was monitored after induction of *cidA* expression in E. coli ΔSR cells containing the plasmids, pS-F-CidA/R^+^ and pS-F-CidA/R^−^. ΔSR cells containing p*S_am7_*/R^+^ and pS-F-minibax/R^−^ were used as negative and positive controls, respectively. (B) Localization of CidA protein to the ΔSR cell membranes was detected by Western blotting using monoclonal anti-FLAG antibodies. ΔSR cells containing p*S_am7_*/R^+^ were used as a negative control since they do not contain a FLAG tag, and pS-F-minibax/R^−^ was used as a positive control. Membrane proteins were separated on a 10% denaturing polyacrylamide gel and stained using monoclonal anti-FLAG antibodies. Data represent the mean ± standard error of the mean (SEM) from three independent experiments, each with three technical replicates.

**FIG 3:**
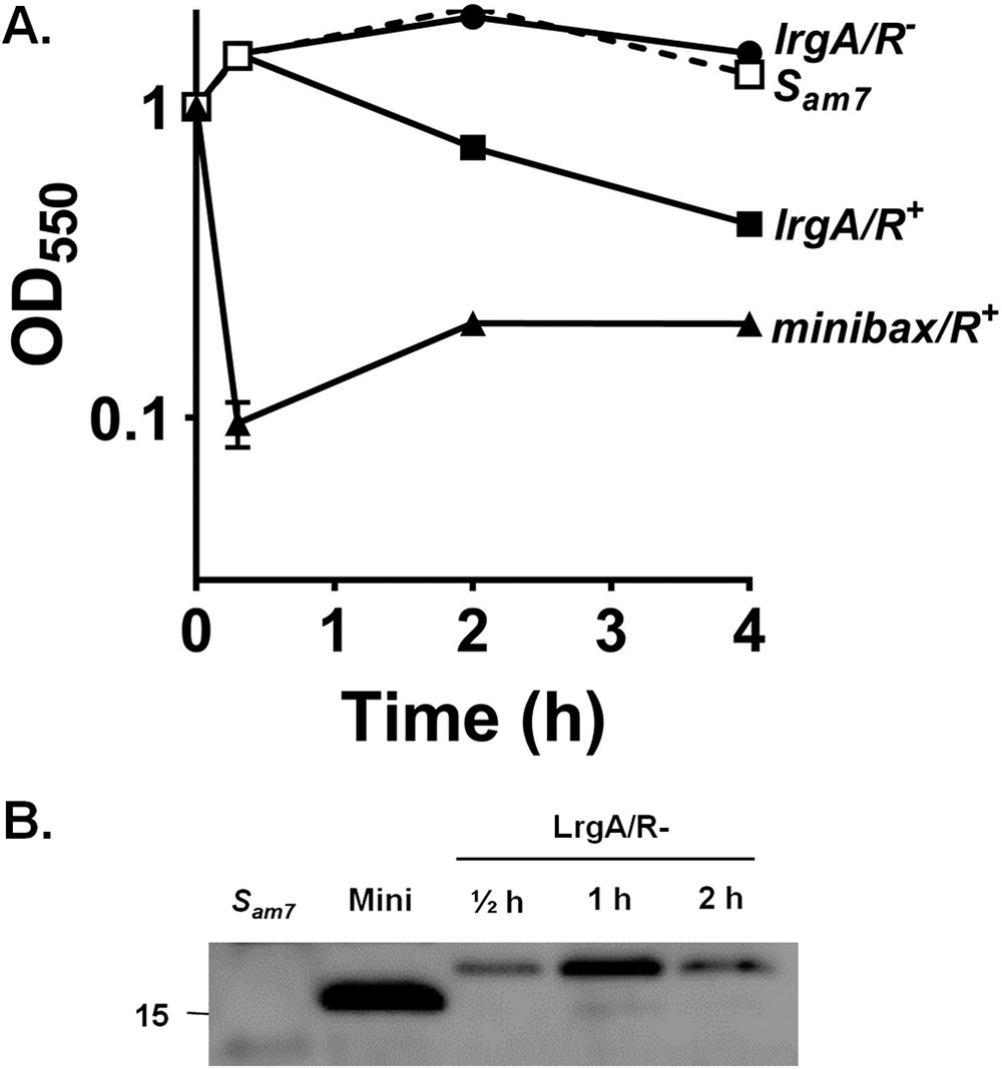
LrgA protein exhibits holin-like properties. (A) Cell lysis was monitored after induction of *lrgA* expression in E. coli ΔSR cells containing the plasmids, pS-F-LrgA/R^+^ and pS-F-LrgA/R^−^. ΔSR cells containing p*S_am7_*/R^+^ and pS-F-minibax/R^−^ were used as negative and positive controls, respectively. (B) Localization of LrgA protein to the ΔSR cell membranes was detected by Western blotting using monoclonal anti-FLAG antibodies. ΔSR cells containing p*S_am7_*/R^+^ (which lacks a FLAG tag) were used as a negative control, and pS-F-minibax/R^−^ was used as a positive control. The protein concentration was adjusted by normalizing the cell amount and assessed as total OD_550_ units collected. Membrane proteins were separated on a 10% denaturing polyacrylamide gel and stained using monoclonal anti-FLAG antibodies. The experiment was repeated three times, and a representative gel image is shown. Data represent the mean ± SEM from three independent experiments, each with three technical replicates.

To determine if CidA and/or LrgA exhibit holin-like activity, we constructed derivatives of pS105 (pSC8 and pSC20), wherein the gene encoding FLAG-tagged CidA or LrgA replaced the *S* gene encoding the S105 holin (Fig. 1A), and transferred these plasmids into E. coli ΔSR cells. Upon thermal induction, the E. coli ΔSR cells underwent a steady rate of cell lysis compared to the holin negative control (*S_am7_*) that lasted over a period of 6 h (Fig. 2A and 3A). As a positive control, we utilized E. coli ΔSR cells containing the plasmid pS-F-minibax/R^+^, which was previously shown to induce lysis in this system (8). To test whether the observed lysis was dependent on endolysin (R), we used an endolysin-negative construct, pSC9 or pSC21 (Fig. 1B), with a stop codon introduced in the *R* gene. As shown in Fig. 2A and 3A, the null mutation in the *R* gene abolished CidA- and LrgA-dependent cell lysis, demonstrating that lysis was not the result of a general toxicity effect of CidA and LrgA. Consistent with this was the demonstration of a time-dependent accumulation of CidA or LrgA within the E. coli ΔSR cytoplasmic membrane (Fig. 2B and 3B). Collectively, these data suggest that cell lysis resulting from heterologous expression of CidA or LrgA in E. coli ΔSR cells is associated with the membrane localization of these proteins, likely resulting in the formation of pores large enough to allow for the passage of the ∼18-kDa *R* gene-encoded endolysin.

### CidA and LrgA form pores within artificial cell membranes.

**FIG 4:**
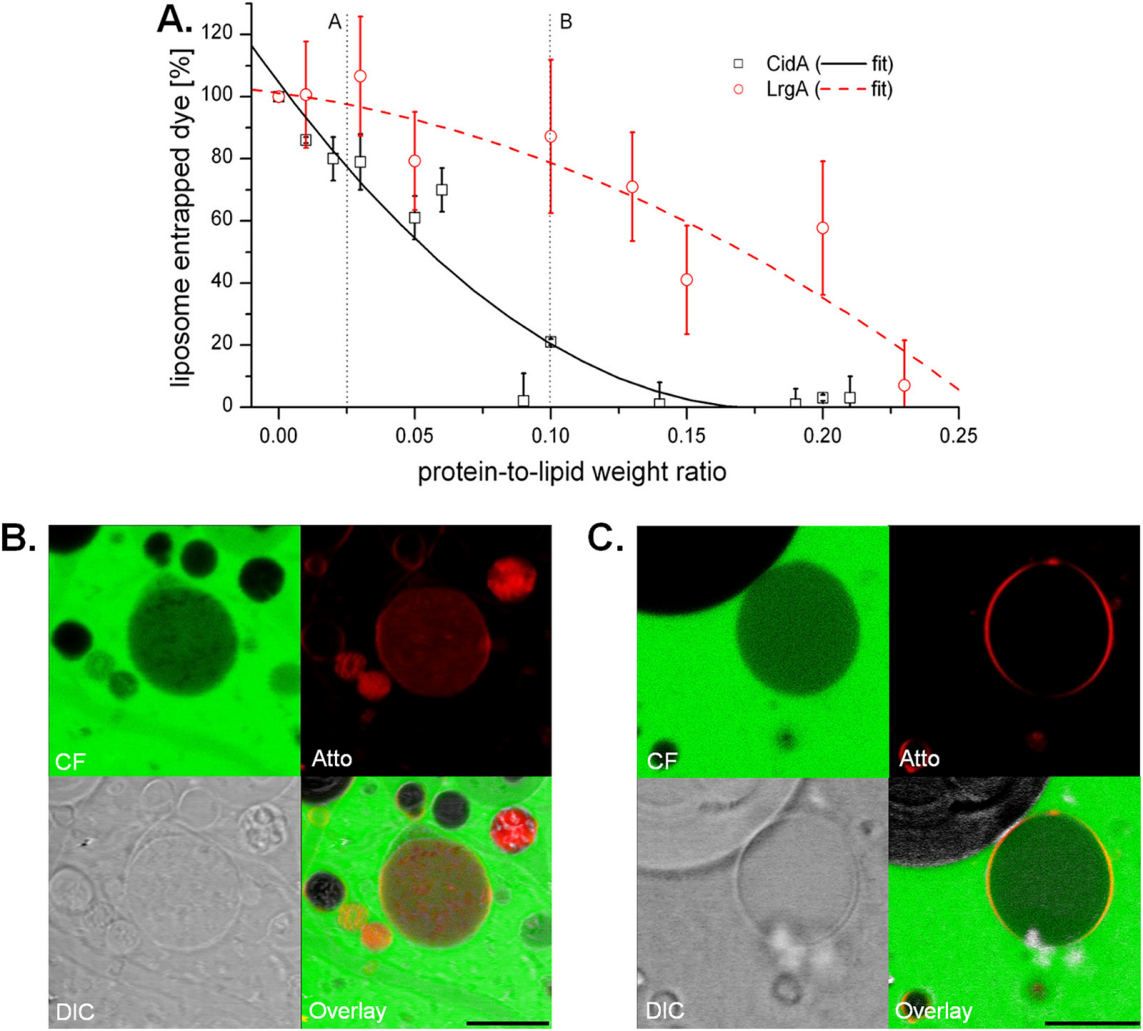
Liposome leakage of carboxyfluorescein in the presence of CidA and LrgA. (A) Liposomes were mixed with purified CidA or LrgA proteins dissolved in DDM and then injected into a solution containing 50 mM CF, 20 mM Tris, 50 mM NaCl, and 2 mM EDTA. As the detergent levels drop, proteoliposomes are formed, trapping the dye inside the interior of the liposomes. Free dye was removed by separation on a PD-10 column, and the collected liposomes were ruptured using 10% Triton X-100 or water, followed by the measurement by spectrometry of the amount of dye released. The amount of liposome-entrapped dye was calculated as ΔFL = FL_Triton X-100_ − FL_water_, where ΔFL represents the difference in fluorescence signal, FL_Triton X-100_ represents the signal obtained from the Triton X-100-treated liposomes (lysed), and FL_water_ represents water-treated liposomes (not lysed). A negative control was made by using plain DDM detergent without proteins. To obtain visual confirmation of leakage, giant unilamellar vesicles (GUVs) were prepared by electroformation with either CidA (B) or LrgA (C), followed by the addition of carboxyfluorescein and the protein stain Atto, and then imaged by confocal microscopy. Confocal image channels are represented as follows: carboxyfluorescein at the top left, Atto at the top right, DIC at the bottom left, and an overlay at the bottom right. The scale bar in the bottom right of the overlay image represents 10 μm.

To provide direct evidence of the ability of CidA and/or LrgA to form a pore, we developed an *in vitro* protein-induced leakage assay using synthetic vesicles. Monomeric CidA-His and LrgA-His were obtained by gel filtration and confirmed by SDS-PAGE and Western blot analysis (see Fig. S1 in the supplemental material). Recombinant CidA-His or LrgA-His was then reconstituted in a mixture of POPG [1-palmitoyl-2-oleoyl-sn-glycero-3-phospho-(1′-rac-glycerol)] and POPC (1-palmitoyl-2-oleoyl-glycero-3-phosphocholine) lipids (7:3) by detergent dialysis as described in Materials and Methods. As previously reported, both of these proteins are predicted to have four transmembrane domains with the N and C termini present on the cytoplasmic face of the cell membrane (14). The localization of the CidA-His or LrgA-His proteins within artificial membrane vesicles was assessed using 5-nm Ni^2+^-gold nanoparticles that specifically bound to the His tag. Transmission electron microscopy (TEM) analysis confirmed the incorporation of CidA-His or LrgA-His within the lipid membranes of artificial vesicles (unpublished data). To determine whether CidA-His and LrgA-His formed functional pores, we loaded the protein-containing vesicles with carboxyfluorescein (CF) (∼376 Da) and assessed dye leakage from the interior of the vesicles. We used vesicles reconstituted with influenza M2, an ion channel transmembrane peptide, as a negative control for our experiments. Influenza M2 allows only the passage of protons and not the large-sized molecules such as CF, even when added at high concentrations (see Fig. S2 in the supplemental material). While CidA-His and LrgA-His both resulted in the leakage of the CF in a concentration-dependent manner, the amount of CidA-His necessary for leakage was much smaller (Fig. 4A). As a way to visualize the leakage of CF, we performed confocal imaging of giant unilamellar vesicles (GUVs) in which the dye was added externally to the vesicles containing either CidA-His or LrgA-His. The externally added CF dye entered vesicles that stained positive for the presence of protein (CidA or LrgA) within the membrane (Fig. 4B and C). Overall, 85% of the CidA-His-containing vesicles and 72% of LrgA-His positive vesicles facilitated entry of the CF dye. In contrast, those vesicles lacking protein (CidA-His or LrgA-His) in their membranes did not allow leakage of the CF dye (unpublished results). Additionally, only approximately 10% of those vesicles prepared without protein allowed for CF dye penetration (see Fig. S3 in the supplemental material). Based on these results, we conclude that both CidA and LrgA promote pore formation within artificial membranes in a concentration-dependent manner.

10.1128/mbio.02827-21.1 FIG S1 Purification of monomeric CidA-His and LrgA-His proteins. Purified CidA-His and LrgA-His proteins were analyzed by performing (A) size-exclusion chromatography of the purified proteins on a Superdex 75 column (arrows indicate the majority of the protein). Proteins were then analyzed by (B) Coomassie-stained SDS-PAGE of purified monomeric CidA-His/LrgA-His and (C) Western blotting of CidA-His/LrgA-His by probing with anti-pentahistidine antibodies conjugated to horseradish peroxidase. Download FIG S1, TIF file, 0.1 MB. Copyright © 2022 Endres et al. 2022 Endres et al. https://creativecommons.org/licenses/by/4.0/ This content is distributed under the terms of the Creative Commons Attribution 4.0 International license.

10.1128/mbio.02827-21.2 FIG S2 Liposome leakage of carboxfluorescein in the presence of influenza M2. As described in the legend to Fig. 4, liposomes were mixed with purified protein (in this case, influenza M2), and then injected into a 50 mM carboxyfluorescein solution. After removal of the free dye, the collected liposomes were ruptured using 10% Triton X-100 or water, followed by the measurement of the amount of dye released by spectrometry. Download FIG S2, TIF file, 0.1 MB. Copyright © 2022 Endres et al. 2022 Endres et al. https://creativecommons.org/licenses/by/4.0/ This content is distributed under the terms of the Creative Commons Attribution 4.0 International license.

10.1128/mbio.02827-21.3 FIG S3 Confocal images of GUVs with no protein. Giant unilamellar vesicles (GUVs) were prepared by electroformation without the addition of purified protein, followed by the addition of carboxyfluorescein and the protein stain Atto. The GUVs were then imaged by confocal microscopy. Confocal image channels are represented as follows: carboxyfluorescein at the top left, Atto at the top right, DIC at the bottom left, and an overlay at the bottom right. The scale bar in the bottom right of the overlay image represents 10 μm. Download FIG S3, TIF file, 0.1 MB. Copyright © 2022 Endres et al. 2022 Endres et al. https://creativecommons.org/licenses/by/4.0/ This content is distributed under the terms of the Creative Commons Attribution 4.0 International license.

### The *lrgAB* operon is required for pyruvate utilization under microaerobic conditions.

**FIG 5:**
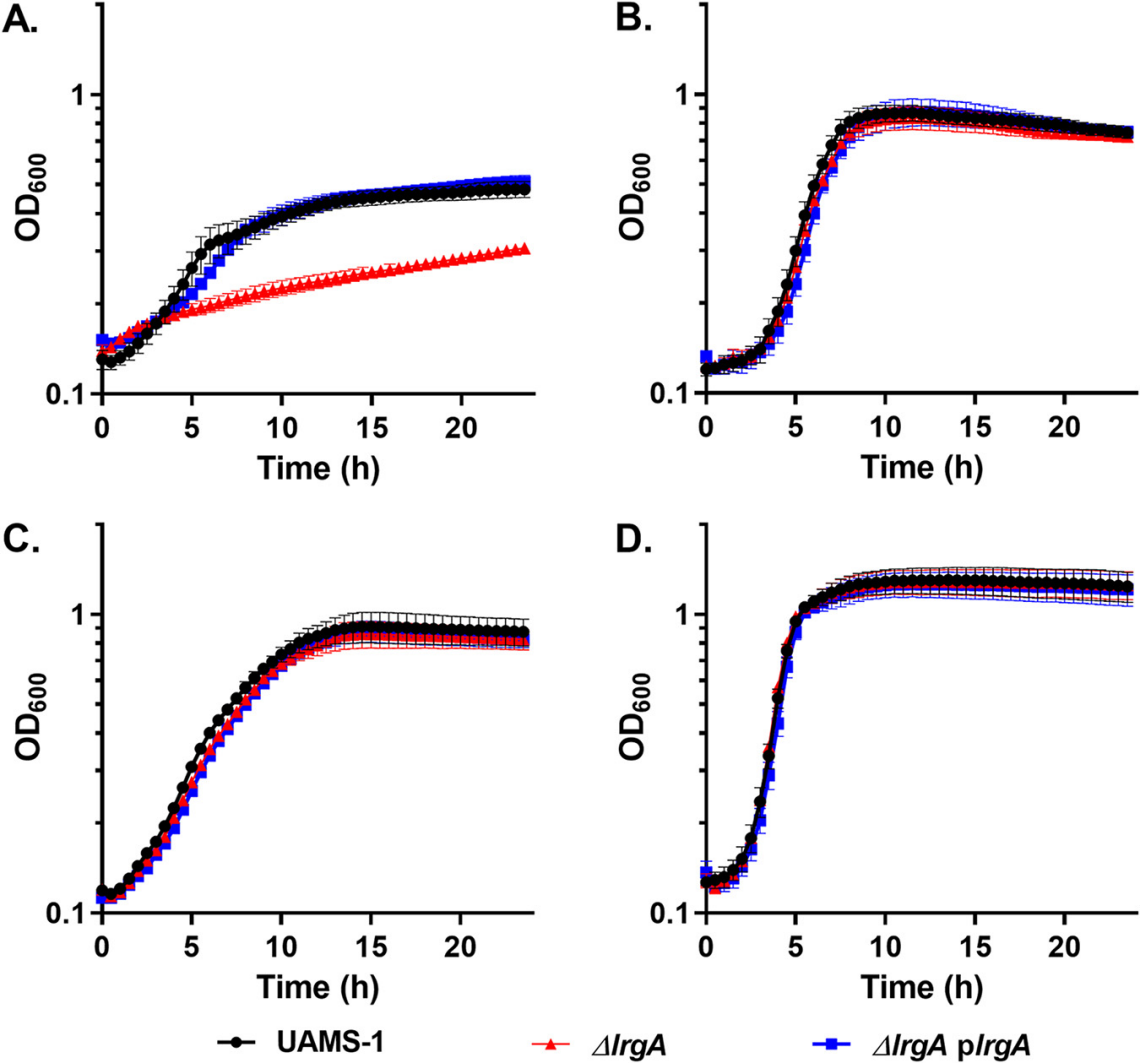
S. aureus *lrgA* is necessary for microaerobic growth with pyruvate as the sole carbon source. Shown is growth of S. aureus UAMS-1 (black circles), UAMS-1 Δ*lrgA* (red triangles), and UAMS-1 Δ*lrgA* with pJE31 (blue squares) in (A) CDM with 30 mM sodium pyruvate grown under microaerobic conditions, (B) CDM with 30 mM pyruvate grown under aerobic conditions, (C) CDM with 15 mM glucose (microaerobic), and (D) CDM with 15 mM glucose (aerobic). Data represent the mean ± SEM from three independent experiments, each with three technical replicates.

**FIG 6:**
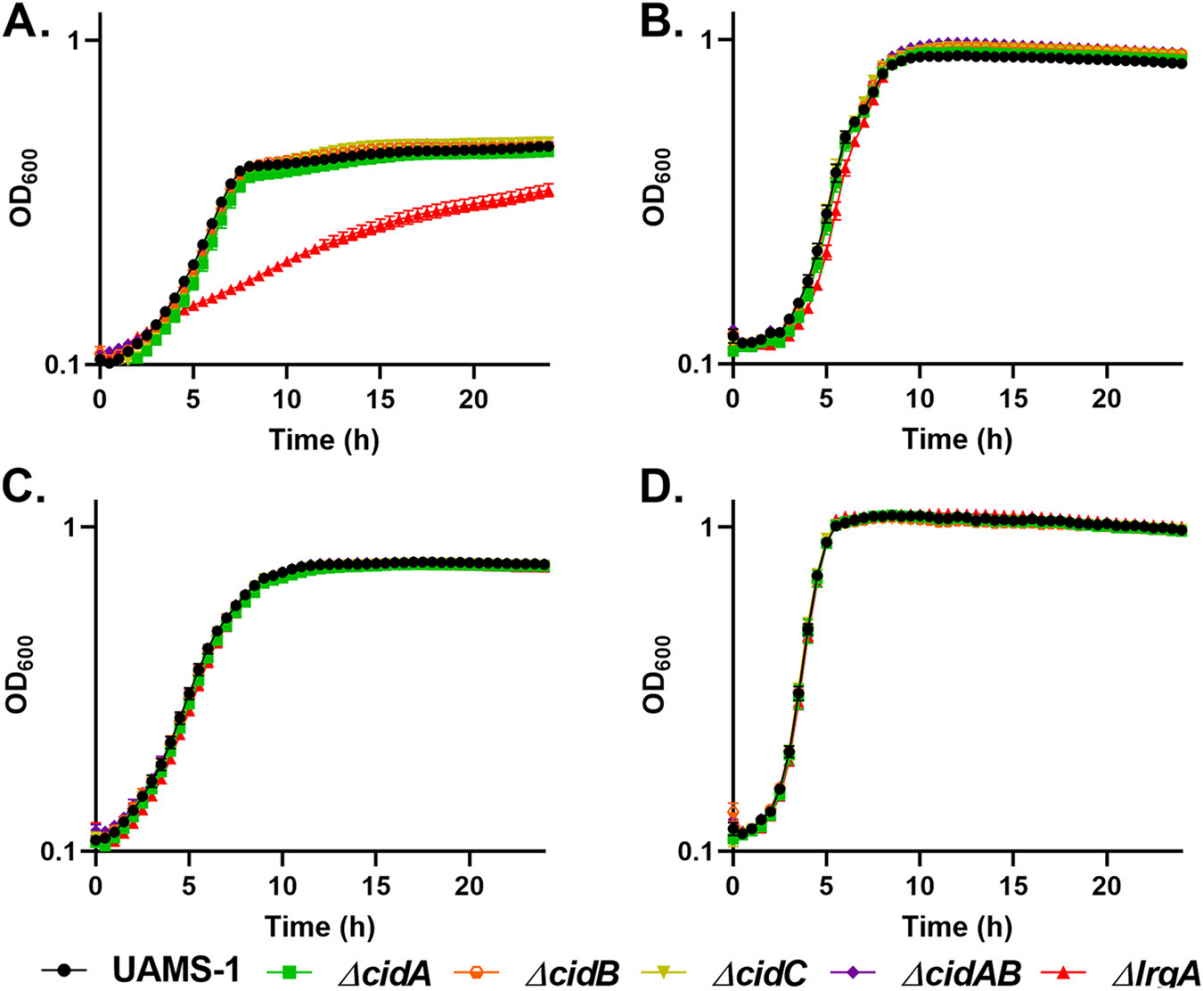
The S. aureus *cid* operon is not required for microaerobic growth with pyruvate as the sole carbon source. Shown is growth of S. aureus UAMS-1 (black circles), UAMS-1 Δ*cidA* (green squares), and UAMS-1 Δ*cidB* (orange circles), UAMS-1 Δ*cidC* (mustard upside-down triangles), UAMS-1 Δ*cidAB* (purple diamonds), and UAMS-1 Δ*lrgA* (red triangles) in (A) CDM with 30 mM pyruvate (microaerobic), (B) CDM with 30 mM pyruvate (aerobic), (C) CDM with 15 mM glucose (microaerobic), and (D) CDM with 15 mM glucose (aerobic). The UAMS-1 Δ*lrgA* mutant was included as a control during all experiments with the *cid* mutants. Data represent the mean ± SEM from three independent experiments, each with three technical replicates.

In light of our previous report that the *cidABC* operon affects export of acetate and acetoin (19), combined with several studies demonstrating a role for *lrgAB* in pyruvate transport in other bacterial species (23–25), an *lrgA* clean deletion mutation was generated, and growth was monitored in chemically defined medium (CDM) containing either 15 mM glucose or 30 mM pyruvate. As shown in Fig. 5, no growth defect was observed when the *lrgA* mutant was grown aerobically with either glucose or pyruvate (Fig. 5B and D). However, when this mutant was grown with pyruvate as the sole carbon source under microaerobic conditions, the *lrgA* mutant exhibited a dramatic growth defect (Fig. 5A) that was not observed when glucose was provided as the sole carbon source (Fig. 5C). In addition, when tested under anaerobic conditions, the *lrgA* mutant exhibited no growth after 24 h with pyruvate as the sole carbon source (unpublished results). Importantly, expression of *lrgA* from a plasmid from its native promoter restored growth of the mutant to wild-type levels (Fig. 5A), confirming the role of this gene under these conditions. To determine if this is a pyruvate-specific phenotype, growth of the *lrgA* mutant was assessed in the presence of other carbon sources (glycerol, fructose, malate, lactate, acetate, and succinate) under aerobic and microaerobic conditions. As shown in Fig. S4 in the supplemental material, although each carbon source supported different growth rates, the *lrgA* mutation did not alter the growth observed in their presence. In contrast, the *cidABC* operon, which was previously shown to affect acetate and acetoin excretion (19), does not affect growth on pyruvate as strains containing mutations within these genes exhibited similar growth patterns to the wild-type strain when using pyruvate as a carbon source (Fig. 6).

10.1128/mbio.02827-21.4 FIG S4 Growth of S. aureus strains on different carbon sources. S. aureus UAMS-1 (black), UAMS-1 Δ*lrgA* (blue), and UAMS-1 Δ*lrgA* with pJE31 (red) were grown aerobically (circles) or microaerobically (triangles) in the presence of different carbon sources: glycerol (A), fructose (B), malate (C), lactate (D), acetate (E), and succinate (F). Data represent the mean ± SEM from three independent experiments with three technical replicates. Download FIG S4, TIF file, 0.5 MB. Copyright © 2022 Endres et al. 2022 Endres et al. https://creativecommons.org/licenses/by/4.0/ This content is distributed under the terms of the Creative Commons Attribution 4.0 International license.

**FIG 7:**
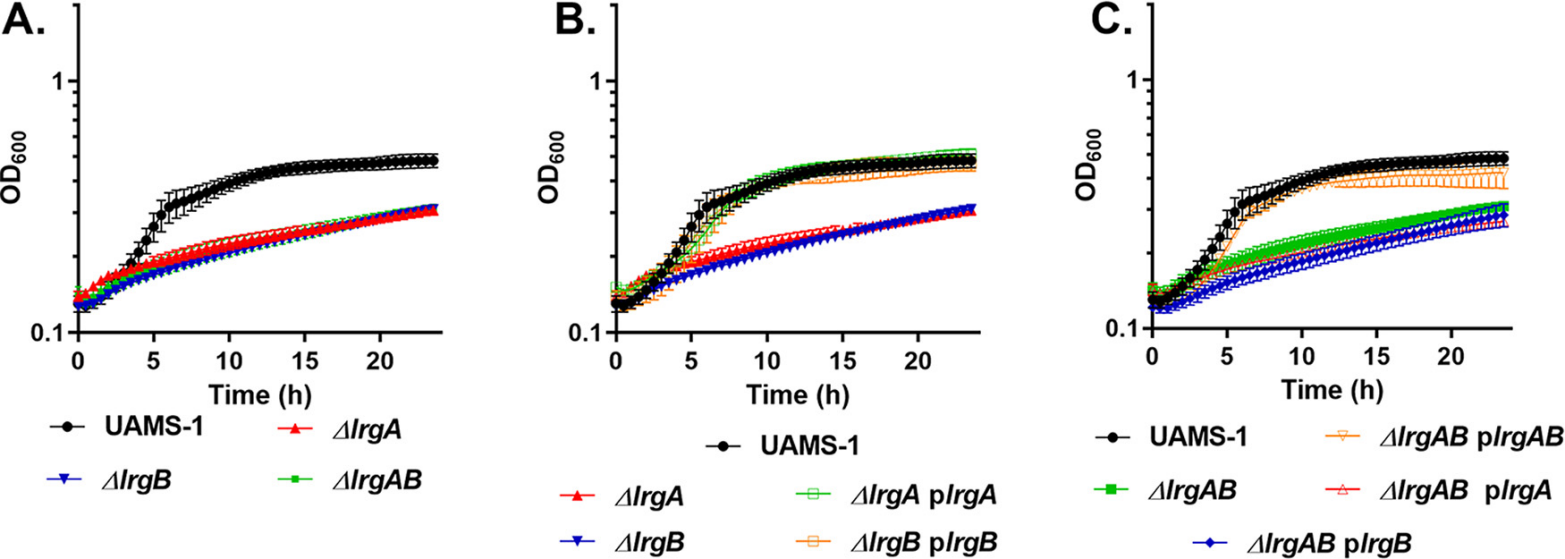
Both *lrgA* and *lrgB* are necessary for microaerobic growth with pyruvate as the sole carbon source. Microaerobic growth in CDM with 30 mM pyruvate of S. aureus. (A) UAMS-1, UAMS-1 Δ*lrgA*, UAMS-1 Δ*lrgB*, and UAMS-1 Δ*lrgAB*. (B) UAMS-1, UAMS-1 Δ*lrgA*, UAMS-1 Δ*lrgB*, and complement strains UAMS-1 Δ*lrgA* containing pJE31 and UAMS-1 Δ*lrgB* with pJE32. (C) UAMS-1, UAMS-1 Δ*lrgAB* and complement strains of UAMS-1 Δ*lrgAB* harboring either pJE30 (*lrgAB*), pJE31 (*lrgA*), or pJE32 (*lrgB*). Data represent the mean ± SEM from three independent experiments, each with three technical replicates.

The *lrgAB* operon consists of two cotranscribed genes, *lrgA* and *lrgB* (28). While LrgA alone has the ability to oligomerize and form pores within the cytoplasmic membrane, resulting in death and lysis, it is likely that this protein carries out these functions in the context of LrgB. Thus, we speculated that disruptions of *lrgB* would also affect growth in the presence of pyruvate. To test this, mutations in *lrgB* and *lrgAB* were generated, and the mutants’ growth was assessed either during microaerobic or aerobic growth in CDM with pyruvate as the primary carbon source. As can be seen in Fig. 7A, the *lrgA*, *lrgB*, and *lrgAB* mutants demonstrated a similar growth defect when grown under microaerobic conditions, suggesting that these isolates are unable to use pyruvate to support growth. The growth defects of the individual mutants can be complemented by expression of the corresponding gene from a plasmid (Fig. 7B and C). However, plasmids carrying either *lrgA* or *lrgB* alone were unable to restore the growth of the *lrgAB* mutant, suggesting that both LrgA and LrgB are necessary for the utilization of pyruvate when grown under microaerobic conditions.

**FIG 8:**
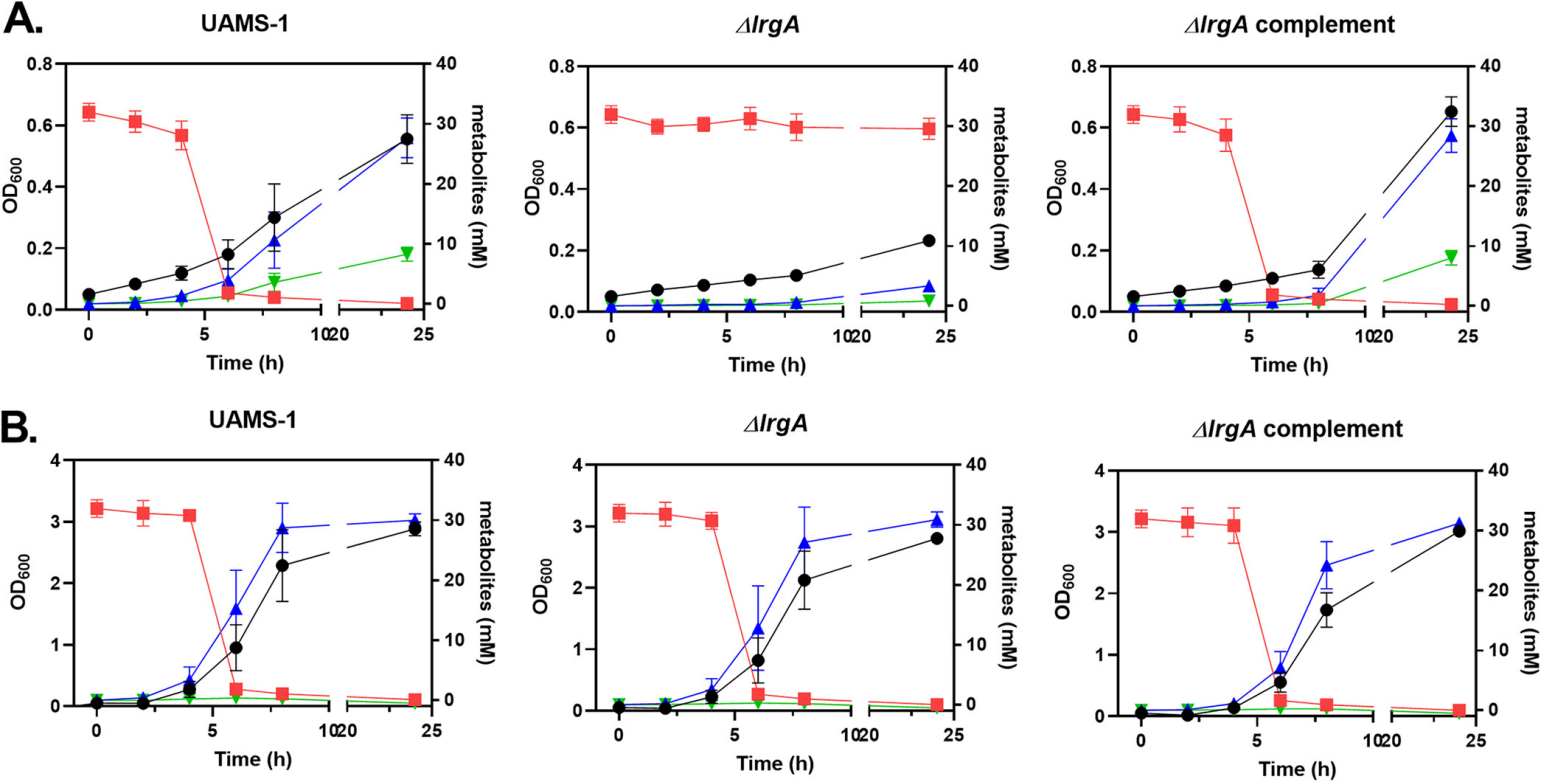
S. aureus *lrgA* is required for pyruvate removal from the media. OD_600_ (black circles), extracellular pyruvate (red squares), acetate (blue triangles), and lactate (green upside-down triangles) were measured at 0, 2, 4, 6, 8, and 24 h for UAMS-1, UAMS-1 Δ*lrgA*, and UAMS-1 Δ*lrgA* containing pJE31 that were grown in CDM with 30 mM pyruvate either (A) microaerobically or (B) aerobically. Data represent the mean ± SEM from three independent experiments, each with three technical replicates.

To assess whether the defect in growth of the *lrgA* mutant in the presence of pyruvate as the sole carbon source was due to its inability to consume pyruvate, the amount of pyruvate remaining in the media after growth was measured for the wild-type, *lrgA* mutant, and complemented strains. As shown in Fig. 8A, growth of the wild-type and complemented strains under microaerobic conditions resulted in the removal of nearly all the pyruvate from the medium by 6 h postinoculation, whereas the *lrgA* mutant was unable to remove any pyruvate even after 24 h of growth. In agreement with the reduced consumption of pyruvate from the media, the *lrgA* mutant also exhibited a dramatic decrease in the amount of lactate and acetate generated during microaerobic growth in the presence of pyruvate compared with the wild-type and complemented strains (Fig. 8A). Consistent with the lack of an effect of the *lrgA* mutation on pyruvate uptake when grown aerobically or with glucose as the primary carbon source, the *lrgA* mutant did not exhibit any differences in the accumulation of lactate and acetate under these conditions compared with the wild-type and complemented strains (Fig. 8B).

### Microaerobic growth with pyruvate induces *lrgAB* expression.

**FIG 9:**
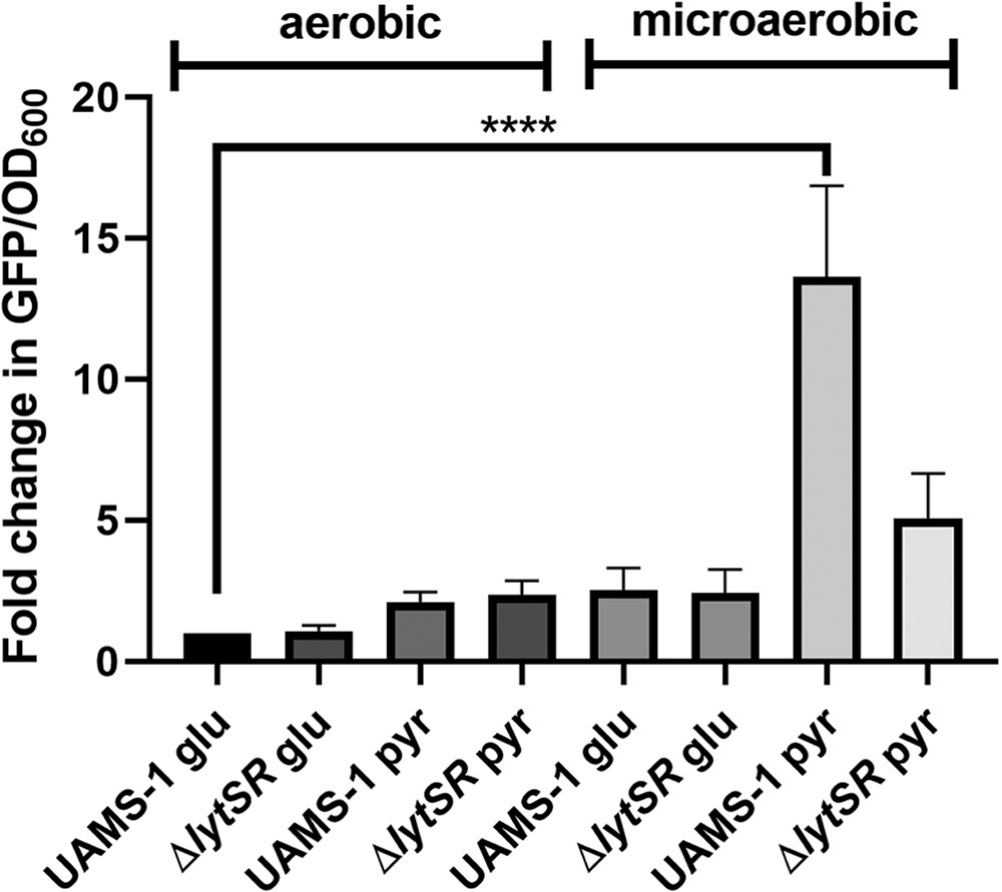
The *lrg* operon is optimally induced when grown under microaerobic conditions with pyruvate as primary carbon source. S. aureus UAMS-1 containing pEM80 was grown in CDM supplemented with either 15 mM glucose or 30 mM pyruvate and with high aeration or under microaerobic conditions. After 6 h of growth, GFP fluorescence was measured using a Tecan M200 Pro microplate reader with excitation at 488 nm and emission collected between 520 and 560 nm. Data represent the mean ± SEM from three independent experiments, each with three technical replicates. Statistical significance was determined by one-way analysis of variance (ANOVA) with a *P* value of <0.0001.

The LytSR two-component regulatory system is necessary for induction of *lrgAB* expression upon disruption of the proton motive force (PMF) (29) and weak acid stress (30). Recent studies have also shown that pyruvate is a potent inducer of *lrgAB* expression in B. subtilis (23) and S. mutans (31). To determine if pyruvate also stimulates S. aureus *lrgAB* expression, a *lrgAB* reporter fusion plasmid, pEM80 (32), was used to monitor *lrgAB* expression under microaerobic conditions in the presence of pyruvate. As shown in Fig. 9, there was a 13-fold increase in green fluorescent protein (GFP) produced by the wild-type strain grown in the presence of pyruvate compared to cells grown aerobically in the presence of glucose. Although some pyruvate-dependent induction of *lrgAB* expression was observed in the *lytSR* mutant, this difference was found not to be statistically significant compared to the expression from the wild-type strain during aerobic growth with glucose as the carbon source. These results demonstrate that S. aureus *lrgAB* expression is induced in an *lytSR*-dependent manner by growth in the presence of pyruvate.

## DISCUSSION

A fundamental hypothesis that has driven our research on the *cidABC* and *lrgAB* operons is that they are important regulators of bacterial PCD and that this mechanism is fundamentally conserved in a wide variety of organisms spanning multiple kingdoms of life. Using a similar “lysis cassette” approach to that described here, we reported that Bax and Bak, the two primary effectors of apoptosis, are functional holins (8). Importantly, 10 regulatory Bcl-2 proteins (similar to Bax and Bak, but playing auxiliary roles) expressed in the lysis cassette system did not induce lysis, revealing a good correlation between Bcl-2 protein function and holin activity. One important aspect of holins is that despite the vast variations in the holin structure and differences in the mode of action of lysis, the end result of their presence is the death and lysis of the bacterial cell (5, 33). Thus, we used the lysis cassette system (Fig. 1) to examine the ability of CidA and LrgA to support cell lysis. As shown in Fig. 2 and 3, both of these proteins supported robust cell lysis, indicative of holin activity. As with other holins tested in this system, lysis was dependent on the presence of endolysin as a drop in optical density was not observed in strains lacking the *R* gene. Consistent with our previous studies (14), the CidA and LrgA proteins were detected in the membrane fractions of the R-negative strains (Fig. 2B and 3B) and that they form high-molecular-weight oligomers (14, 15). Interestingly, we routinely observed regrowth of the minibax control (Fig. 2 and 3) and speculate that this is a result of the accumulation of mutations that confer resistance of the cells to lysis by minibax or are simply the result of plasmid curing. Why regrowth was not observed with the CidA and LrgA expression constructs was not determined but is the subject of current investigation. Finally, the hole-forming potential of CidA and LrgA was supported using liposomes reconstituted with purified CidA and LrgA, which induced leakage of carboxyfluorescein (Fig. 4). While both CidA and LrgA induced carboxyfluoroscein leakage, CidA did so at a much lower protein-to-lipid ratio than LrgA (Fig. 4A), suggesting that its ability to form holes is more efficient. Although these results alone do not rule out the possibility that these proteins induce a more general membrane destabilization, combined with the biological data presented in Fig. 2 and 3, it is concluded that CidA and LrgA function through the generation of pores.

Bacteriophage-encoded holins are an incredibly diverse family of proteins, with more than 52 recognized families identified (34). Although there are a few exceptions, holins are characterized by their relatively small size, the presence of 1 to 4 transmembrane domains, hydrophobicity, and their ability to oligomerize within the cytoplasmic membrane. Currently, there are two models of holin function, the canonical holin-endolysin and the pinhole-“SAR” endolysin system, both of which result in a precisely timed lysis of the cell by allowing an enzyme with muralytic activity to gain access to the peptidoglycan. The canonical holin-endolysin system generates pores in the membrane that are nonspecific and vary in size, with an average hole size of roughly 340 ± 35 nm (35), while the SAR endolysin model generates small 2-nm channels. Both systems cause the collapse of the proton motive force (PMF), allowing either for the release of cytosolic endolysin or for a membrane-embedded SAR endolysin to fold into an active conformation (36). Given the size of the endolysin (∼18 kDa) released by the lysis cassette (1, 2) used in this study, we speculate that CidA and LrgA function in a manner similar to the canonical holin-endolysin system.

Although our previous work on the CidA and LrgA proteins has been focused on their roles in bacterial PCD, it has become increasingly clear that they also serve as transporters of the by-products of carbohydrate metabolism (19, 37). The first evidence of this was the observation that the *cidABC* operon is important for regulating bacterial cell death during overflow metabolism. Specifically, when excess glucose is present the *cidA* and *cidB* mutants exhibited opposing roles in the excretion of acetate and acetoin (19), the former likely due to the interaction of *cidA* or *cidB* with *cidC*, coding for a pyruvate:menaquinone oxidoreductase that uses pyruvate as its substrate to generate acetate (21). More recently, several studies have demonstrated the role of *lrgAB* homologs in the transport of pyruvate (23–25). In B. subtilis, disruption of the *lrgAB* homologs (designated *ysbAB*) caused a significant growth defect when grown in LB media with pyruvate as the primary carbon source (23). Similarly, the S. mutans *lrgAB* operon also appears to play a role in pyruvate import. Although S. mutans is unable to grow with pyruvate as the sole carbon source, an *lrgAB* mutant was defective in pyruvate consumption upon entry into stationary phase (25). Interestingly, the plant *lrgAB* homolog *PLGG1* has been shown to be important modulator of cell death (38), as well as being involved in the transport of glycerate and glycolate during photorespiration (22).

To determine the potential role of the S. aureus *lrgAB* operon in pyruvate transport, we first examined the effect of a *lrgA* mutation on growth in the presence of this metabolite. Consistent with previous reports of metabolite transport, the *lrgA* mutant exhibited a growth defect when grown under microaerobic conditions in the presence of pyruvate (Fig. 5 and 6). In addition, the *lrgA* mutant was unable to remove the pyruvate from the media during microaerobic growth (Fig. 8). This phenotype was not observed under aerobic conditions, suggesting the presence of another pyruvate transporter in the presence of oxygen. Since the *cidABC* operon had previously been shown to affect metabolite transport, *cidA*, *cidB*, and *cidC* mutants were also tested under the same conditions. As shown in Fig. 6, no differences in growth were observed, indicating that this operon does not affect the utilization of pyruvate. Thus, the results presented in this report demonstrate that the S. aureus *cidA* and *lrgA* genes encode a unique class of holins that are involved in the transport of small metabolic by-products of carbohydrate metabolism. Based on our knowledge of how holins form pores (5, 27, 35, 39), we speculate that the *cidA-* and *lrgA-*encoded holins mediate import and export and have no selectivity for different molecules below a certain size. However, it is important to keep in mind that for the membrane vesicle experiments, *cidA* and *lrgA* were studied in the absence of their natural partners, *cidB* and *lrgB*, respectively, and are therefore likely functioning out of context of their normal molecular environment. In agreement with this, plasmids carrying either *lrgA* or *lrgB* alone were unable to complement the *lrgAB* mutant during microaerobic growth with pyruvate as the primary carbon source, suggesting that both components of the operon are required under these conditions. Based on these results, we speculate that LrgA and LrgB form a complex within the membrane that functions in the capacity of a pyruvate transporter and that LrgB provides specificity for pyruvate.

Based on the results generated by this study and others (19, 21, 40), one could speculate that these two operons work in concert to transport pyruvate into the cell for the substrate of the gene products of the *cid* operon. However, testing of extracellular acetate and acetoin levels under carbon overflow conditions (35 mM) revealed that the *lrgA* mutation does not affect the product of these metabolites (unpublished results). One caveat of these experiments is that the amount of acetate generated during overflow metabolism conditions by the Pta-AckA pathway overshadows the effects of the *cidABC* or *lrgAB* mutations on acetate production. Thus, we generated the *cidABC* mutations in a *pta*/*ackA* mutant background and were able to demonstrate the effects of the *cidABC* mutations on acetate production (19). However, we have been unable to generate either *lrgA*/*ackA* or *lrgA*/*pta* mutants to date. One possible reason for this is that disruptions of *pta* and *ackA* result in toxic levels of pyruvate in the cytoplasm (41) and the *lrgAB* operon is important for regulating the amount of pyruvate within the cell. In eukaryotic cells, the amount of pyruvate within the mitochondria is known to be tightly regulated, as too much or too little of this important metabolite leads to a disease state (42). In fact, molecules that block the import of pyruvate into the mitochondria are being used to treat these illnesses, including Parkinson’s disease and diabetes (43). In this respect, there are conflicting reports on whether the *lrgAB* homologs have the ability to act as a pyruvate exporter as well. In Bacillus subtilis, pyruvate can be exported via the *lrgAB* homologs, but they do not appear to play this role in Streptococcus mutans (23, 25). The ability of the S. aureus *lrgAB* operon to export pyruvate remains to be determined and is the subject of our current investigations.

## MATERIALS AND METHODS

### Bacterial strains, plasmids, and growth conditions.

The bacterial strains used for this study are listed in Table S1 in the supplemental material. For convenience, we have referred to the MC4100 [λΔ(*SR*)] strain as ΔSR. ΔSR cells carrying pS105-derived plasmids were grown in lysogeny broth (LB) medium supplemented with 100 μg/mL ampicillin. Lysis curves were obtained by growing the ΔSR cells aerobically (250 rpm) at 30°C until an optical density at 550 nm (OD_550_) of ∼0.3 was reached, followed by thermal induction for 20 min at 42°C. Incubation was continued at 37°C for 6 h to observe the impact of induced gene expression on lysis (OD_550_) and viability (CFU/mL).

10.1128/mbio.02827-21.5 TABLE S1 Strains and plasmids used in this study. Download Table S1, DOCX file, 0.03 MB. Copyright © 2022 Endres et al. 2022 Endres et al. https://creativecommons.org/licenses/by/4.0/ This content is distributed under the terms of the Creative Commons Attribution 4.0 International license.

S. aureus strains were grown overnight in tryptic soy broth (TSB) medium (BD Biosciences) at 37°C with aeration at 250 rpm. For growth analyses, the overnight cultures were pelleted and resuspended into chemically defined medium (CDM) that was prepared as previously described (44) with either pyruvate or glucose to a final concentration of 30 or 15 mM, respectively. Cultures were then inoculated to a final OD_600_ of 0.05, and growth was monitored by OD_600_ measurements taken every 30 min using a Tecan plate reader. For microaerobic growth in the Tecan plate reader, the wells were covered with 2 drops of MitoXpress HS mineral oil (Agilent Technologies) to reduce the diffusion of oxygen in the liquid medium.

For the metabolite analysis, bacterial cultures were incubated in CDM as described above, except they were inoculated into a 250-mL flask. Aerated samples were grown with a 1:10 volume to flask ratio at 37°C and shaking at 250 rpm, while microaerobic samples were grown with a 3:5 volume-to-flask ratio with shaking at 50 rpm.

### Examination of holin function.

The holin-like functions of *cidA* and *lrgA* were tested by cloning these genes into the pS105 derivatives pS-Fxe-miniBax/R^+^ and pS-GFP/R^−^. Both of these plasmids were designed to express N-terminal FLAG-tagged effectors upon induction by heat (8). To initiate the cloning process, the pS105-based vectors were digested with XhoI and EcoRI to obtain the pS105 vector backbones containing R^+^ and R^−^ endolysin alleles, but devoid of minibax or GFP. Next, the *cidA* gene was PCR amplified using cidA XhoI_F and cidA EcoRI_R primers, digested with XhoI and EcoRI, and ligated with the gel-purified pS105/R^+^ and pS105/R^−^ backbones. The resulting constructs were referred to as pSC8 and pSC9. In a separate experiment, the *lrgA* gene was amplified using the primers lrgA XhoI_F and lrgA EcoRI_R, and ligated into pS105 using a similar approach to *cidA*. The resulting constructs were designated pSC20 and pSC21. All four cloned constructs were transformed into E. coli ΔSR for assessment of holin function.

### ΔSR cell membrane preparation and Western blotting.

Cultures of ΔSR cells transformed with p*S_am7_*/R^−^, pS-F-miniBax/R^−^, pS-F-cidA/R^−^, or pS-F-lrgA/R^−^ plasmids were grown in 50 mL of LB medium supplemented with 100 μg/mL ampicillin to an OD_550_ of ∼0.3 at 30°C, induced for 15 min at 42°C, and then grown at 37°C. Samples were collected after 30 min, 1 h, and 2 h and then were passed through an EmulsiFlex-C5 three times at 10,3421.36 kPa of pressure. The membranes were collected by ultracentrifugation at 100,000 × *g* for 60 min at 4°C and resuspended in 200 μL of 1× phosphate-buffered saline (PBS) containing proteinase inhibitor (Roche, catalog no. 11836170001). For Western blotting, resuspended membrane fractions were mixed with 3× SDS sample buffer, subjected to the SDS-PAGE, and probed with an anti-FLAG antibody (Sigma, catalog no. F3165).

### Protein expression and purification.

For the purification of C-terminal His-tagged CidA and LrgA (designated CidA-His and LrgA-His, respectively), the *cidA* and *lrgA* expression plasmids pDR7 and pDR8, respectively, were transferred into E. coli strain C43, a mutant derivative of BL21(DE3) (14). E. coli C43 cells containing plasmid were grown at 37°C (200 rpm) in tryptone-yeast extract medium (2× TY) supplemented with 0.1 mg/mL kanamycin. After the cultures reached an OD_600_ of ∼3.0, they were cooled to 27°C and induced with 1 mM isopropyl-β-d-1-thiogalactopyranoside (IPTG) until an OD_600_ of ∼5.0 to 6.0 was reached. Cells were then harvested by centrifugation using a Beckman S-5.1 rotor at 5,000 rpm for 10 min at 4°C and stored at −20°C. The frozen cells were lysed by adding 2.5 mM Triton X-100 (critical micelle concentration [CMC], 0.2 to 0.9 mM), 0.8 M urea, 0.25 mg/mL lysozyme, and Pierce universal nuclease (Thermo Fisher Scientific), followed by stirring at room temperature for 1 h. Total membrane solubilization was achieved by the addition of 65 mM Empigen BB detergent (Sigma-Aldrich) (CMC, 1.5 mM) and further incubation at room temperature for 1 h. Insoluble material was removed by centrifugation at 7,500 × *g* and 4°C for 1 h. Protein purification was performed using an AKTA Purifer 10 system (GE Healthcare). First, the total cell solubilized material was loaded onto a 20-mL HisPrep FF 16/10 column (GE Healthcare) preequilibrated using a buffer containing 20 mM Tris, 25 mM Empigen BB, 3 M urea, 500 mM NaCl, and 60 mM imidazole (pH 8.0). The column was then washed with 200 mL of the same buffer followed by 200 mL of a buffer containing 20 mM Tris, 0.17 mM *n*-dodecyl β-d-maltoside (DDM) (CMC, 0.17 mM) and 60 mM imidazole (pH 8.0). Second, CidA/LrgA was eluted from the Ni resin using a buffer containing 20 mM Tris, 0.17 mM DDM, and 500 mM imidazole (pH 8.0) and then directly applied to a 25-mL Superdex 200 Increase 10/300 column (GE Healthcare) for a second-step purification in 20 mM Tris–0.17 mM DDM buffer (pH 8.0). Purified protein was stored at −20°C in the presence of 20% glycerol.

### Protein characterization.

Purified CidA-His and LrgA-His were analyzed by SDS-PAGE using Coomassie blue staining and by Western blotting using an anti-pentahistidine antibody conjugated to horseradish peroxidase. Samples were boiled at 95°C for 10 min before loading.

Separation of monomeric, unfolded CidA-His/LrgA-His was accomplished first by precipitation of the octyl-β-glucoside-(OG)-solubilized protein with methanol-chloroform and resolubilization in neat formic acid. The sample was then diluted with FMA (20% each of formic acid, methanol, and acetonitrile in water) and separated by size exclusion chromatography on a Superdex 75 column (GE Healthcare) in FMA at a flow rate of 1 mL/min. The fractions corresponding to monomeric CidA/LrgA were collected and used immediately. This sample was used to determine the CidA-His/LrgA-His molecular weight by direct injection into an electrospray ionization quadrupole time-of flight (ESI-Q-TOF) mass spectrometer (Waters) and the CidA-His/LrgA-His N-terminal amino acid sequence by lyophilization and subsequent Edman degradation using a Procise 494 protein sequencer (Applied Biosystems) at the UNMC proteomics facility.

### Membrane reconstitution and liposome generation.

CidA-His/LrgA-His in DDM buffer was reconstituted by detergent dialysis into a membrane system comprised of the phospholipids POPG [1-palmitoyl-2-oleoyl-sn-glycero-3-phospho-(1′-rac-glycerol)] and POPC (1-palmitoyl-2-oleoyl-glycero-3-phosphocholine) (Avanti Polar Lipids), at a ratio of 7:3. Lipids were dissolved in chloroform and stored at −20°C. Aliquots containing the required amount of lipids were placed in Eppendorf tubes, and chloroform was removed under low vacuum followed by overnight lyophilization. The dried lipid mixtures were then dissolved in 20 mM Tris–60 mM OG (pH 8.0) (CMC, 20 to 25 mM) at a final concentration of 2.5 mg/mL and vortexed until clear. OG-solubilized lipids and CidA-His or LrgA-His were then combined at a 1:8 ratio (wt/wt) and incubated at room temperature for 30 min. To form liposomes, the mixture was dialyzed using a 10-kDa-cutoff membrane against a mixture of 20 mM Tris, 50 mM NaCl, and 2 mM EDTA for 24 h. The amount of protein, if any, that becomes embedded in the liposomes is dependent on the speed at which the detergent is removed from the mixture (45).

### Carboxyfluorescein liposome leakage assays.

The liposome leakage assay was based on the self-quenching property of 5(6)-carboxyfluorescein (CF) (Acros Organics), a negatively charged fluorescent dye. The POPG and POPC lipids (at a 7:3 ratio) were dried and dissolved in 60 mM OG as described above using the dialysis-based membrane reconstitution method. CidA/LrgA proteins dissolved in DDM (CMC, ∼0.17 mM), as well as a high concentration of CF (50 mM in 20 mM Tris, 50 mM NaCl, and 2 mM EDTA), which self-quenches at this concentration, were added to the lipid-protein detergent mixture, diluting the detergent, allowing the liposomes to form and, thus, trapping the CF. Free dye and the proteoliposomes were separated by PD-10 columns, and the collected liposomes were treated with 10 μL of 10% Triton X-100 or water, respectively. Upon lysis with Triton X-100, the CF entrapped inside the liposomes was immediately diluted and emitted a fluorescent signal, which was measured using a spectrometer at an endpoint excitation wavelength 492 nm and emission wavelength 517 nm. The signal was calculated as ΔFL = FL_Triton X-100_ − FL_water_. ΔFL represents the difference in fluorescence signal, FL_Triton X-100_ represents the signal obtained from the Triton X-100-treated liposomes (lysed), and Fl_water_ stands for water-treated liposomes (not lysed). A negative control was created by generating liposomes in buffer containing lipids and CF but lacking CidA-His and LrgA-His. By comparing the ΔFL of CidA-His/LrgA-His proteoliposomes and the control liposomes, the extent of leakage induced by the membrane-entrapped protein was calculated.

### Confocal imaging of entrapped CF.

Protein-embedded giant unilamellar vesicles (GUVs) were generated by electroformation (46) in an indium tin oxide (ITO)-coated glass chamber connected to the Vesicle Prep Pro (Nanion Technologies). Briefly, 1 mg/mL dipalmitoylphosphatidylcholine (DPPC)-cholesterol that was dissolved in chloroform was deposited on the ITO-coated glass surface, and the solvent was allowed to evaporate, leaving the lipids in a dehydrated lamellate phase. An O-ring was then placed around the dried lipid, and 300 μL of PBS was carefully added to the lipid film. A second ITO-coated slide was then placed on top of the O-ring, with the ITO layer facing downwards. Electroformation was controlled by a Vesicle Prep Pro with an alternating voltage of 3 V peak to peak being applied with a progressive increase for the rise time and a frequency of the alternating current of 5 Hz. Directly after electroformation, purified CidA-His or LrgA-His was mixed with the GUVs and incubated at room temperature for 1 h. The protein stain nitrilotriacetic acid (NTA)-Atto 647N (Sigma-Aldrich) was used to visualize any CidA-His/LrgA-His protein present. CF was then added, and liposome leakage was imaged using a Zeiss 710 confocal scanning microscope fitted with an EC Plan-Neofluar 40×/1.30 oil (differential inference contrast [DIC]) M27 objective. CF was excited with an argon 458/488/514-nm laser, while the NTA-Atto 647N was excited using a diode-pumped solid-state (DPSS) 561-nm laser.

### Generation of the *lrgA*, *lrgB*, and *lrgAB* mutants and complement strains.

The *lrgA* mutation in UAMS-1 was generated by allelic exchange as previously described, utilizing the temperature-sensitive plasmid pCL52.2 (47). First, approximately 1,000 bp of the upstream region of *lrgA* was PCR amplified using the primers JBLRGA1.2 and JBLRGA2. The PCR product was digested with EcoRI and XhoI, gel purified, and ligated into EcoRI/XhoI-digested pCL52.2. The resulting plasmid was named pJB27. Next, roughly 1,000 bp of the downstream region of *lrgA* was PCR amplified using the primers JBLRGA3 and JBLRGA4, and the resulting PCR product was digested with PstI and XhoI and ligated to PstI/XhoI-digested pJB27 to generate the plasmid pJB30. To generate the *lrgB* mutant, primers JBLRGB1 and JBLRGB4, which added EcoRI and PstI sites, were used to amplify the entire *lrgAB* operon, along with approximately 500 bp upstream of the *lrgA* start codon and 1,000 bp downstream of the *lrgB* stop codon. This fragment was ligated into pCR2.1 (Invitrogen), generating pJB2. To remove the XhoI site from pJB2, the plasmid was digested with XhoI, Klenow treated, and then self-ligated, generating the plasmid pJB10. Inverse PCR was performed on pJB10 using the primers JBLRGB2.2 and JBLRGB3, which added XhoI sites allowing for self-ligation and resulted in the removal of *lrgB*. Finally, the fragment from the self-ligated plasmid containing 941 bp upstream of *lrgB* and 1,023 bp downstream of *lrgB* was ligated into pCL52.2 at the HindIII/XbaI sites, generating pJB14. Finally, to generate the *lrgAB* mutant, a 1,042-bp region downstream of *lrgB* was amplified using the primers JBLRGB3 and JBLRGB4 (which added PstI sites) and then ligated into PstI-digested pCL52.2. The resulting plasmid was named pJB4. The upstream region of *lrgA* was then amplified using the primers JBLRGA1.2 and JBLRGA2, creating an ∼1,000-bp fragment containing EcoRI and XhoI restriction sites that were used to ligate the fragment to the similarly digested pJB4, creating the plasmid pJB27. All of these plasmids were then electroporated into RN4220 and moved into UAMS-1 via Φ11-mediated bacteriophage transduction (48). Following confirmation of the presence of each plasmid, allelic exchange was carried out as previously described (49), and the mutations were confirmed by PCR amplification. The primers JBLRGA4 and JBLRGA5 were used to confirm the *lrgA* mutation, the *lrgB* mutant was confirmed using the primers JBLRGA5 and JBLRGB6, and the *lrgAB* mutant was confirmed using the JBLRGB1.2 and JBLRGB4 primers.

The *lrgA* complementation plasmid was generated by PCR amplification of ∼500 bp upstream of the *lrgA* start site and ending at the *lrgA* stop codon, using the lrgAB-comp-F and lrgA-comp-R primers, which added EcoRI and BamHI restriction sites to the product. The resulting 1,003-bp product was digested and ligated into EcoRI- and BamHI-digested pLI50, generating the plasmid pJE31. The *lrgAB* and *lrgB* complementation plasmids were generated using the lrgAB-comp-F and lrgAB-comp-R primers. UAMS-1 wild-type DNA was used as the template for the *lrgAB* amplification, while the *lrgA* mutant was utilized as the template for *lrgB*. The PCR fragments were then digested and ligated into the EcoRI/BamHI sites of pLI50 and named pJE30 and pJE32, respectively. The plasmids were then electroporated into RN4220 and moved into the appropriate strain by Φ11-mediated bacteriophage transduction.

### Metabolite analysis.

For the pyruvate analysis, 1-mL samples were collected from bacterial cultures every 2 h for a total of 8 h and then again at 24 h of growth. The bacteria were pelleted, 900 μL of supernatant was transferred to a clean 1.5-mL tube, and samples were frozen at −20°C. The pyruvate assay was performed as previously described (50, 51). Briefly, samples were diluted and added to a black 96-well plate with a clear bottom and brought to a 50-μL total volume with the pyruvate assay buffer (100 mM potassium phosphate, 1 mM EDTA, and 1 mM MgCl_2_). Then, 150 μL of an enzyme mix containing 10 μM flavin adenine dinucleotide, 0.2 mM thiamine pyrophosphate, 0.2 U/mL pyruvate oxidase, 0.2 U/mL horseradish peroxidase, and 50 μM Amplex Red in the assay buffer was added to each well and mixed by pipetting. The plate was incubated for 15 min at room temperature in the dark, and then the OD_570_ was measured using a Tecan microplate reader. A standard curve from 0 to 10 mM was generated and used to calculate the amount of pyruvate per well.

The amounts of extracellular acetate and lactate were calculated using an acetic acid test kit (R-biopharm, catalog no. 10148261035) and a d-lactic acid/l-lactic acid test kit (R-biopharm, catalog no. 11112821035), respectively, per the manufacturer’s instructions, but adjusted to be performed in a 96-well format. Briefly, 10 μL of culture supernatant was added to the wells of a 96-well microtiter plate (Corning, catalog no. 3596), followed by the addition of the master mix (313 μL for acetate and 216 μL for lactate) and then read at 340 nm following a 15- or 30-min incubation, respectively, at room temperature.

10.1128/mbio.02827-21.6 TABLE S2 Primers used in this study. Download Table S2, DOCX file, 0.01 MB. Copyright © 2022 Endres et al. 2022 Endres et al. https://creativecommons.org/licenses/by/4.0/ This content is distributed under the terms of the Creative Commons Attribution 4.0 International license.
