# Supplementary figures and images for: The Staphylococcus aureus CidA and LrgA Proteins Are Functional Holins Involved in the Transport of By-Products of Carbohydrate Metabolism
Source: mBio. 2022 Feb 1;13(1):e02827-21. doi: 10.1128/mbio.02827-21 (PMC8805020; doi:10.1128/mbio.02827-21)

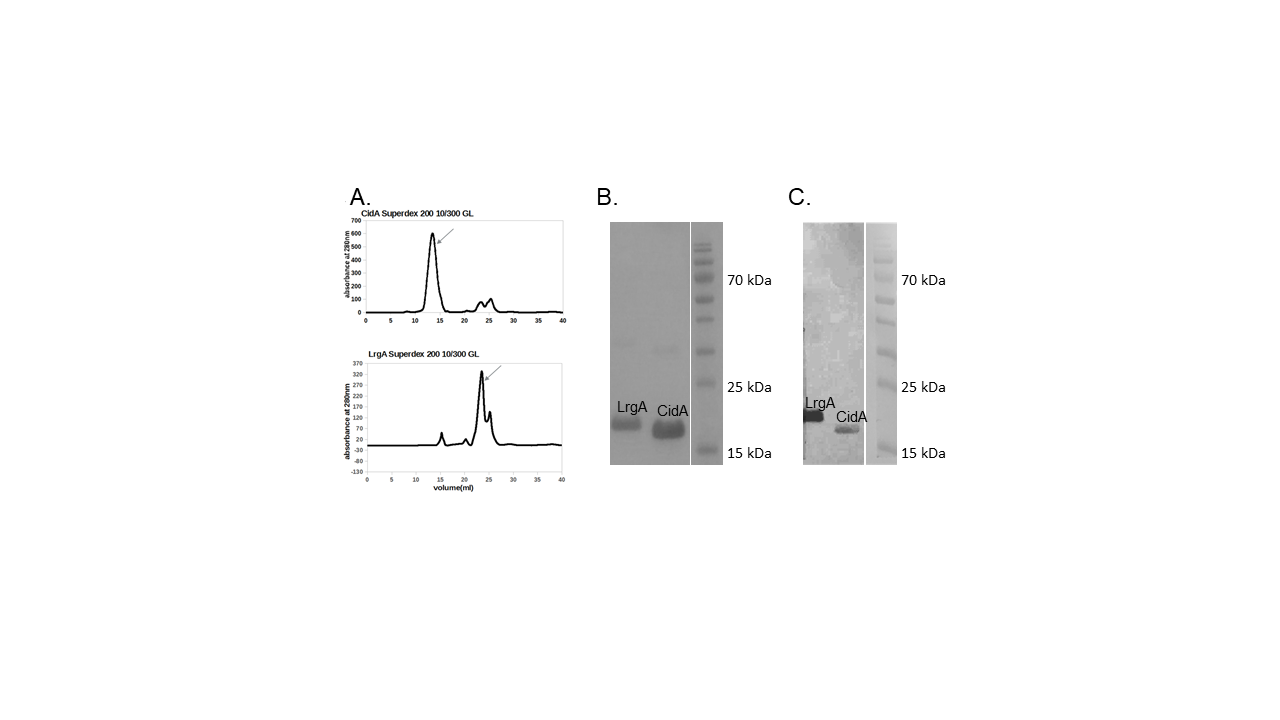

Supplement: FIG S1 [file mbio.02827-21-sf001.tif]

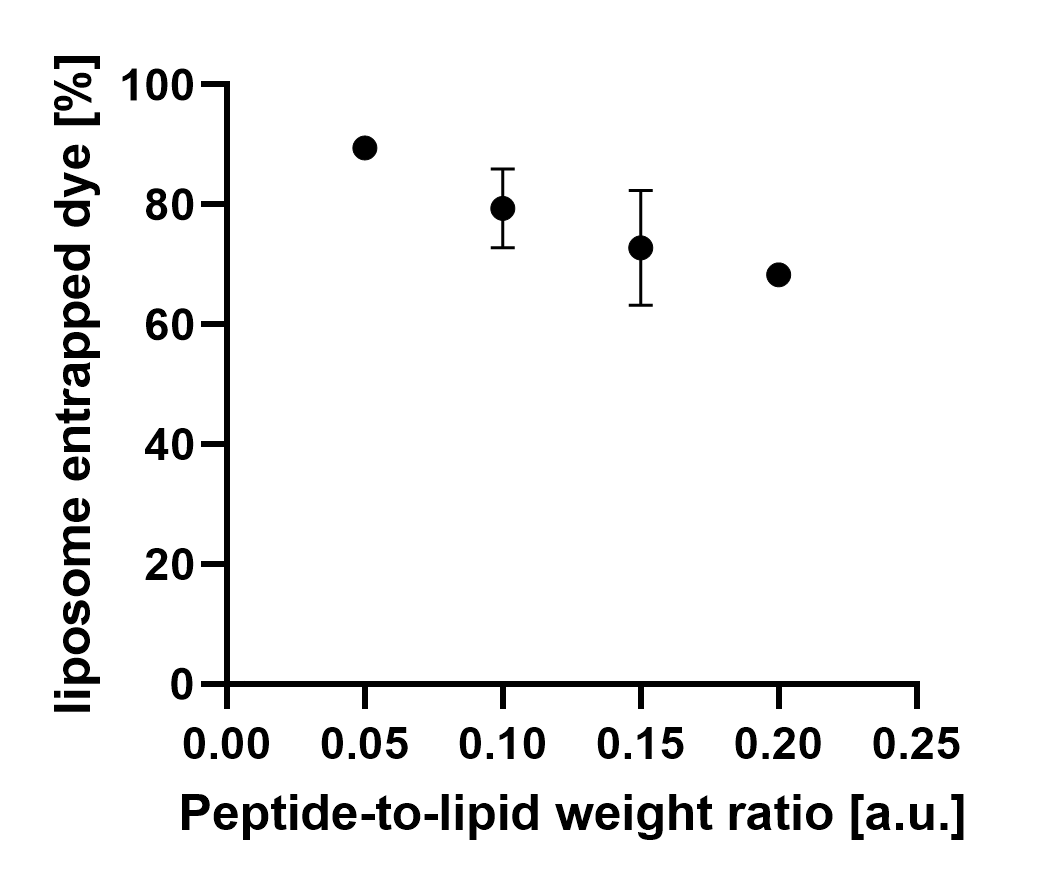

Supplement: FIG S2 [file mbio.02827-21-sf002.tif]

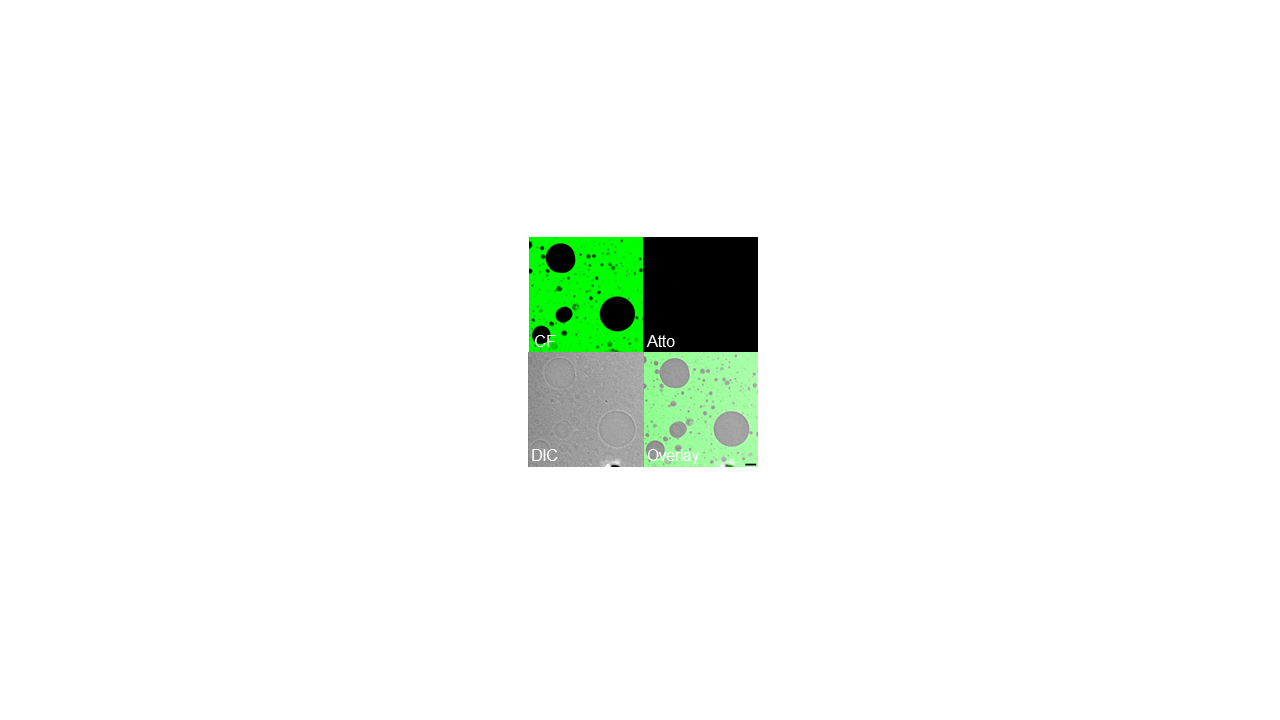

Supplement: FIG S3 [file mbio.02827-21-sf003.tif]

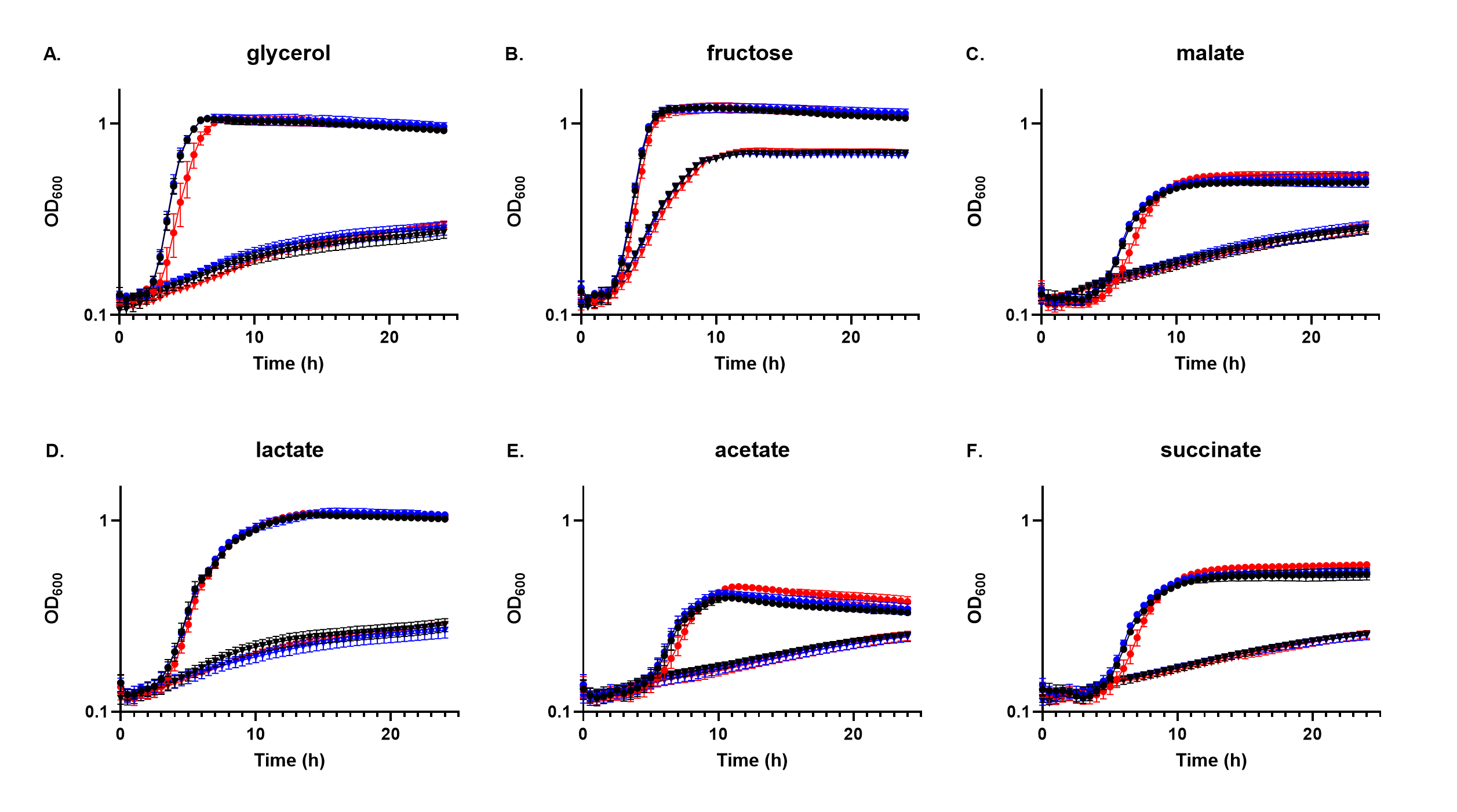

Supplement: FIG S4 [file mbio.02827-21-sf004.tif]
