# Supplementary material for: The Staphylococcus aureus CidA and LrgA Proteins Are Functional Holins Involved in the Transport of By-Products of Carbohydrate Metabolism
Source: mBio. 2022 Feb 1;13(1):e02827-21. doi: 10.1128/mbio.02827-21 (PMC8805020; doi:10.1128/mbio.02827-21)
Supplement: TABLE S1 [file mbio.02827-21-st001.docx]

| **Table 1. Strains and Plasmids used in this study** | | |
| --- | --- | --- |
| **Bacterial strain or Plasmid** | **Relevant properties** | **Reference** |
| **Strains** |  |  |
| *E. coli* |  |  |
| DH10B |  |  |
| C43 | Derivative of BL21(DE3) for expression of membrane proteins | [1] |
| MC4100 | [λΔ(SR)] | [2] |
|  |  |  |
| *S. aureus* |  |  |
| UAMS-1 | clinical osteomyelitis isolate | [3] |
| KB1065 | UAMS-1 *ΔcidA* | [4] |
| KB1060 | UAMS-1 *ΔcidB* | [4] |
| KB1058 | UAMS-1 *ΔcidC::erm/* Em^R^ | [5] |
| KB1063 | UAMS-1 *ΔlrgA* | This study |
| KB1061 | UAMS-1 *ΔlrgB* | This study |
| KB1064 | UAMS-1 *ΔlrgAB* | This study |
|  |  |  |
| **Plasmids** |  |  |
| pLI50 | *E. coli*- *S. aureus* shuttle vector | [6] |
| pJE30 | pLI50::*lrgAB* (under control of its native promoter) | This study |
| pJE31 | pLI50::*lrgA* (under control of its native promoter) | This study |
| pJE32 | pLI50::*lrgB* (under control of its native promoter) | This study |
| pDR7 | pET24b with CidA-H | [7] |
| pDR8 | pET24b with LrgA-H | [7] |
| pS105 | λ *trans* activation plasmid with S105 holin | [2] |
| pS-Fxe-miniBax/R+ | pS105 endolysin positive altered with FLAG-tag and XhoI/EcoRI cloning sites | [8] |
| pS-GFP/R- | pS105 endolysin negative altered with FLAG-tag and XhoI/EcoRI cloning sites | [8] |
| pS-F-miniBax | pS105/R+ with FLAG-tagged miniBax | [8] |
| p*S_am7_* | Lysis defective S allele | [9] |
| pSC8 | pS-Fxe-miniBax/R+ with *cidA* | This study |
| pSC9 | pS-GFP/R- with *cidA* | This study |
| pSC20 | pS-Fxe-miniBax/R+ with *lrgA* | This study |
| pSC21 | pS-GFP/R- with *lrgA* | This study |
|  |  |  |
|  |  |  |

**References**

1. Miroux, B. and J.E. Walker, *Over-production of proteins in Escherichia coli: mutant hosts that allow synthesis of some membrane proteins and globular proteins at high levels.* J Mol Biol, 1996. **260**(3): p. 289-98.

2. Smith, D.L., et al., *Purification and biochemical characterization of the lambda holin.* J Bacteriol, 1998. **180**(9): p. 2531-40.

3. Gillaspy, A.F., et al., *Role of the accessory gene regulator (agr) in pathogenesis of staphylococcal osteomyelitis.* Infect Immun, 1995. **63**(9): p. 3373-80.

4. Windham, I.H., et al., *SrrAB Modulates Staphylococcus aureus Cell Death through Regulation of cidABC Transcription.* J Bacteriol, 2016. **198**(7): p. 1114-22.

5. Patton, T.G., et al., *The Staphylococcus aureus cidC gene encodes a pyruvate oxidase that affects acetate metabolism and cell death in stationary phase.* Molecular Microbiology, 2005. **56**(6): p. 1664-1674.

6. Lee, C.Y., S.L. Buranen, and Z.H. Ye, *Construction of single-copy integration vectors for Staphylococcus aureus.* Gene, 1991. **103**(1): p. 101-5.

7. Ranjit, D.K., J.L. Endres, and K.W. Bayles, *Staphylococcus aureus CidA and LrgA proteins exhibit holin-like properties.* J Bacteriol, 2011. **193**(10): p. 2468-76.

8. Pang, X., et al., *Active Bax and Bak are functional holins.* Genes Dev, 2011. **25**(21): p. 2278-90.

9. Gründling, A., M.D. Manson, and R. Young, *Holins kill without warning.* Proc Natl Acad Sci U S A, 2001. **98**(16): p. 9348-52.
