# Supplementary material for: The Staphylococcus aureus CidA and LrgA Proteins Are Functional Holins Involved in the Transport of By-Products of Carbohydrate Metabolism
Source: mBio. 2022 Feb 1;13(1):e02827-21. doi: 10.1128/mbio.02827-21 (PMC8805020; doi:10.1128/mbio.02827-21)
Supplement: TABLE S2 [file mbio.02827-21-st002.docx]

**Table 2. Primers used in this study**

| Primer name | Sequence (5'-3') |
| --- | --- |
| lrgAB_comp_F | CCACGAATTCAAACGTATTGAACAAGCAGTC |
| lrgA_comp_R | TTTTGGATCCGGTGTGTTTAGTGCTAAGTGG |
| lrgAB_comp_R | TTTTGGATCCGCTATTATCTTGCTTAGGTTTTTCG |
| JBLRGA1.2 | CCGAATTCAGAATCTGGAACTGGTAGTGC |
| JBLRGA2 | CCCTCGAGTTACACGACCATTGCCTCCTACGTTTG |
| JBLRGA3 | CCCTCGAGCCAGCCGGTATCTCAGTTGTTAACTC |
| JBLRGA4 | GGCTGCAGCGTCTATATCCCAGTTATAAACCGGAG |
| JBLRGA5 | GGTGTCAAGATGCAAGTTGGACGTTC |
| JBLRGB1 | CCGAATTCGCGACTAAAGCCAAAGATGATAATAGCGCA |
| JBLRGB1.2 | CCCCGAATTCGCGACTAAAGCCAAAGATGATAATAGCGCA |
| JBLRGB2.2 | CCCGGCTCGAGGTTAATCATGAGCTTGTGCCTCCTC |
| JBLRGB3 | CCCTCGAGTAAAACGAAAAACCTAAGCAAGATAATAGC |
| JBLRGB4 | GGCTGCAGCCTGCATCCACATCGTATGGCC |
| JBLRGB6 | GGACTAGTCCCAGTTATAAACCGGAGTATAGACG |
| cidA XhoI_F | GATCCTCGAGATGCACAAAGTCCAATTAATAA |
| cidA EcoRI_R | GATCGAATTCTCATTCATAAGCATCTATACCTTT |
| lrgA  XhoI_F | GATCCTCGAGATGGTCGTGAAACAACAAAA |
| lrgA  EcoRI_R | GATCGAATTCTTAATCATGAGCTTGTGCCT |
|  |  |
